# The relationships of serum vitamin D concentration with linear speed and change of direction performance in soccer players

**DOI:** 10.3389/fnut.2024.1501643

**Published:** 2024-11-22

**Authors:** M. M. Michalczyk, M. Kałuża, G. Zydek, R. Roczniok, A. Golas

**Affiliations:** Institute of Sport Sciences, The Jerzy Kukuczka Academy of Physical Education in Katowice, Katowice, Poland

**Keywords:** vitamin D, soccer (football), speed – strength qualities, change of direction ability, muscle

## Abstract

The aim of the study was to establish whether the level of 25 hydroxyvitamin D (25(OH)D) in serum has an influence on speed (m/s) and change of direction (COD, s) performance. Twenty male soccer players from the top league participated in the study. All subjects were evaluated for the serum concentration of 25(OH)D at the beginning of the preseason. The linear sprint test was performed at 5 m and 30 m, and COD (time and deficit) at the beginning (BPP) and after (APP) 6 weeks of the preparatory period. The results revealed that 20% of soccer players had a significant deficiency of 25(OH)D (<20 ng/mL) and 30% had insufficient 25(OH)D levels (between 20 and 30 ng/mL). Positive correlations were found between the training effect for the ∆COD (BPP-APP) (*p* = 0.003) and ∆deficit (BPP-APP) (*p* = 0.039). Significant differences were noticed for the ∆COD (m = 0.60 [s]) and ∆deficit (m = 0.56[s]) in the soccer players whose 25(OH)D concentration was <=30 ng/mL, and for the ∆COD (*p* = 0.002) and ∆deficit (*p* = 0.017) in the soccer players whose 25(OH)D concentration was >30 ng/mL. The training effect was significantly higher for the soccer players whose 25(OH)D concentration was above 30 ng/mL. Soccer players with higher 25(OH)D levels achieved superior results in the COD test and demonstrated better deficit outcomes, affirming the positive influence of 25(OH)D on muscle metabolism.

## 1 Introduction

Over the years, numerous studies have been conducted on the effects of vitamin D on the human body (1–3). Previous research has revealed its significant impact on calcium-phosphate metabolism of the body’s skeletal system (2, 4, 5). However, the discovery of the vitamin D receptor (VDR), which mediates the biological effect of vitamin D, has transformed the understanding of this vitamin (6, 7). 25 hydroxyvitamin D (25(OH)D) is a fat-soluble vitamin that, when converted into its biologically-active form, 1,25-dihydroxyvitamin, influences the expression of over 900 genes (6, 8, 9). These genes impact a wide variety of health and performance aspects, such as the immune and endocrine systems, exercise-induced inflammation, cardiovascular health, cancer prevention, neurological function, glucose metabolism, as well as bone and muscle structure and metabolism (3, 6, 8, 10, 11). Vitamin D also has a significant impact on mental health, including depression, cognitive disorders, and neurological impairments (1, 3, 12). The crucial impact of vitamin D on muscle cells results from its effects on calcium homeostasis, energy metabolism, phospholipid metabolism, cell proliferation and differentiation, protein synthesis, and mitochondrial function (1, 6, 13, 14). Vitamin D also plays an important role in the regulation of skeletal muscle tone and contraction, and it is necessary for a high oxygen consumption rate, muscle strength, power, and preventing muscle weakness and fatigue (15–20). Vitamin D also regulates the synthesis of testosterone and insulin-like growth factor-1, which critically influence muscle structure and function (21–23).

The primary source of vitamin D, aside from the diet which provides small amounts, is skin synthesis during exposure to solar ultraviolet (UVB) radiation (6, 20, 22). Skin synthesis supplies over 90% of the vitamin to the body (6, 24). Small amounts of this vitamin can be obtained from the diet by consuming fatty fish, egg yolks, mushrooms, or dairy products (25). Unfortunately, despite two sources from which vitamin D can be replenished in the body, most inhabitants of countries located at an altitude of 35 N are diagnosed with deficiencies of this vitamin, especially in autumn, winter, and early spring when the daily dose of sunlight is low (1). On the other hand, recent studies revealed that inhabitants of equatorial countries, where daily sunshine is high all year round, are also diagnosed with vitamin D deficiency (9). This discovery was a huge revelation.

According to the latest standards for serum vitamin D, a concentration below 20 ng/mL indicates deficiency, between 20 to 30 ng/mL is considered insufficiency, between 30 to 50 ng/mL is categorized as sufficient, and between 50 to 100 ng/mL is deemed optimal (26). Concentrations above 100 ng/mL indicate toxicity (26, 27). By applying these standards, a number of different authors confirm that vitamin D deficiency in athletes can reach even 60–90% (9, 19, 28–30), especially in athletes who live at or above 35° latitude and train indoors or outdoors but use sunscreen in the summer and protect themselves from the cold in autumn and winter, putting them at risk for serum 25(OH)D insufficient levels or deficits (9, 19, 22). Among soccer players from England, Spain, and Poland, deficits were observed in more than 50% (9, 22, 31–33). The greatest surprise was the results of serum vitamin D levels in soccer players from the Middle East. In Qatar, where daily sunshine is very high, over 80% of 342 soccer players were diagnosed as deficient in vitamin D (34). Interestingly, even during strenuous training, a decline in vitamin D levels was recorded (35). This decrease was significant, although training was performed outdoors in the summer and early autumn months. In athletes, low levels of vitamin D, lower than 30 ng/mL, may decrease anaerobic and aerobic performance and increase frequent injuries and infections (9, 19). It has been hypothesized that a serum 25(OH)D level above the normal reference range (up to 50 ng/mL) could induce beneficial adaptations in skeletal muscle, such as enhanced aerobic performance, both strength and power production, and recovery (15, 36–39).

Soccer is a team sport where aerobic and anaerobic capacity, muscular strength, and speed are important factors for most of the actions during the match (22, 40–42). Due to the great demands of the game, soccer players must possess numerous skills to perform repetitive activities such as sprinting, jumping, accelerating, decelerating, changing direction (COD), which are interspersed with low to medium intensity, e.g., walking or jogging (43, 44). Running with high speed in a straight line and in different directions is particularly important, and during the match, these abilities enable soccer players to make decisions faster (22, 42, 44). Speed and COD abilities require a rapid application of force. According to the latest research, vitamin D deficiency may reduce the ability of muscles to generate force (15). Soccer players who are deficient in vitamin D showed a reduction in performance (22). Studies have shown a correlation between serum 25(OH)D concentration, speed and power efficiency in young soccer players, and speed, endurance, and muscle strength in adult professional players (15, 20, 42, 45–47).

The relationship between vitamin D and muscle performance in soccer players has been studied by several researchers (17, 19, 22, 32, 45, 47, 48). Some researchers confirm its influence (19, 22, 45), while others do not (32, 48). Koundourakis et al. (45) observed a positive correlation between vitamin D levels and muscle performance in a cohort of Greek football players. Michalczyk et al. (22) also confirmed such a correlation. Similarly, a randomized study performed by Close et al. (19) showed the beneficial effects of vitamin D on muscle strength and power, as well as better sprint and vertical jump test results. In contrast, Hamilton et al. (17) found no significant association between serum 25(OH)D levels and muscle function. Similarly, Bezuglov et al. (32) did not confirm such a correlation. Additionally, in a study by Jastrzebska et al. (48), higher vitamin D levels in serum after supplementation did not significantly affect the measured performance parameters of soccer players.

Therefore, two aims of this study were: first of all, to determine the level of 25(OH)D in serum in professional soccer players during winter, and secondly, to evaluate whether the serum level of 25(OH)D affects the speed and change of direction performance (COD) in soccer players on two different occasions, prior to the beginning and at the end of the winter preparatory period. Additionally, we examined whether soccer players whose vitamin D level was below 30 ng/mL, i.e., insufficient concentrations, and those whose level was above 30 ng/mL, i.e., sufficient concentrations, achieved the same results in these tests. We hypothesized that a higher 25(OH)D serum concentration positively correlates with soccer players’ sprint and COD results.

## 2 Materials and methods

### 2.1 Study design

**Figure 1:**
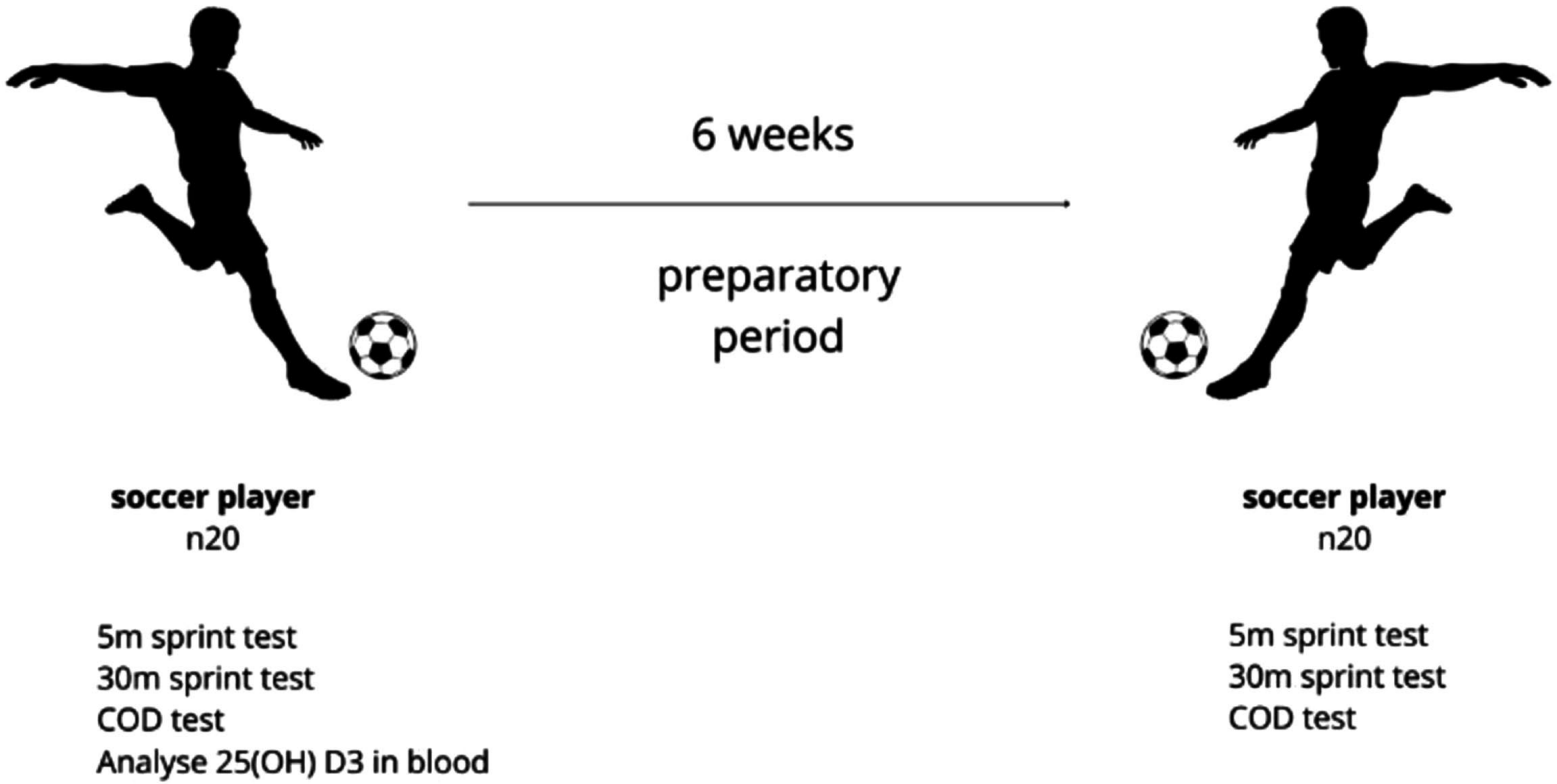
Diagram illustrating the course of the study.

The experiment lasted for 6 weeks and covered the preparation period for the summer season (Figure 1). The athletes trained on an everyday basis (approximately 2 h/d) with an official soccer game on Saturday/Sunday. Additionally, twice a week, the players performed a strength and conditioning training session. After the end of the 6-weeks training period, a test protocol identical to the baseline one was administered. The participants who received vitamin D supplements 30 days or less prior to blood sampling or suffered from acute respiratory viral infections or any other diseases that resulted in absence from three or more training sessions 30 days or less prior to the examination were excluded from the study. The players were advised to adhere to their usual dietary routines throughout the study and refrain from the consumption of any supplements or stimulants throughout the experiment. Additional eligibility criteria to participate in the study were outlined as follows: (a) a minimum of 10 years of training experience, (b) participation in a first-league team, (c) absence of injuries in the 6 months leading up to the assessments, and (d) consistent engagement in training sessions a minimum of 5 times per week over the last 6 months. All participants were informed verbally and in writing about the procedures, possible risks and benefits of the tests and provided written consent before the commencement of the study. All research procedures were reviewed and approved by the bioethical committee of the Academy of Physical Education in Katowice (ethic references KB-05/2017, December 5, 2017); furthermore, the study conformed to the tenets of the Declaration of Helsinki for medical research involving human subjects.

The experimental sessions were conducted between 8:00 and 10:00 a.m. The session was preceded by a warm-up protocol, which included 5 min of cycling, 5 min of treadmill running, several upper and lower body exercise with targeted DROM Exercises (Dynamic Range of Motion Exercise) (43), followed by 5 m and 30 m sprints, COD tests (90°) and two sets of running with submaximal speed. All sprint tests were performed on an indoor field with an artificial grass surface.

### 2.2 Subjects

Twenty male elite soccer players from the First Polish League (age = 22.8 ± 8.2 years, body mass = 75.1 ± 15.3 kg, body height = 178.3 ± 13.5 cm, soccer training experience = 12 ± 3.4 years) took part in this study. The participants were all full-time professionals who trained daily.

### 2.3 Sprint test

**Figure 2:**
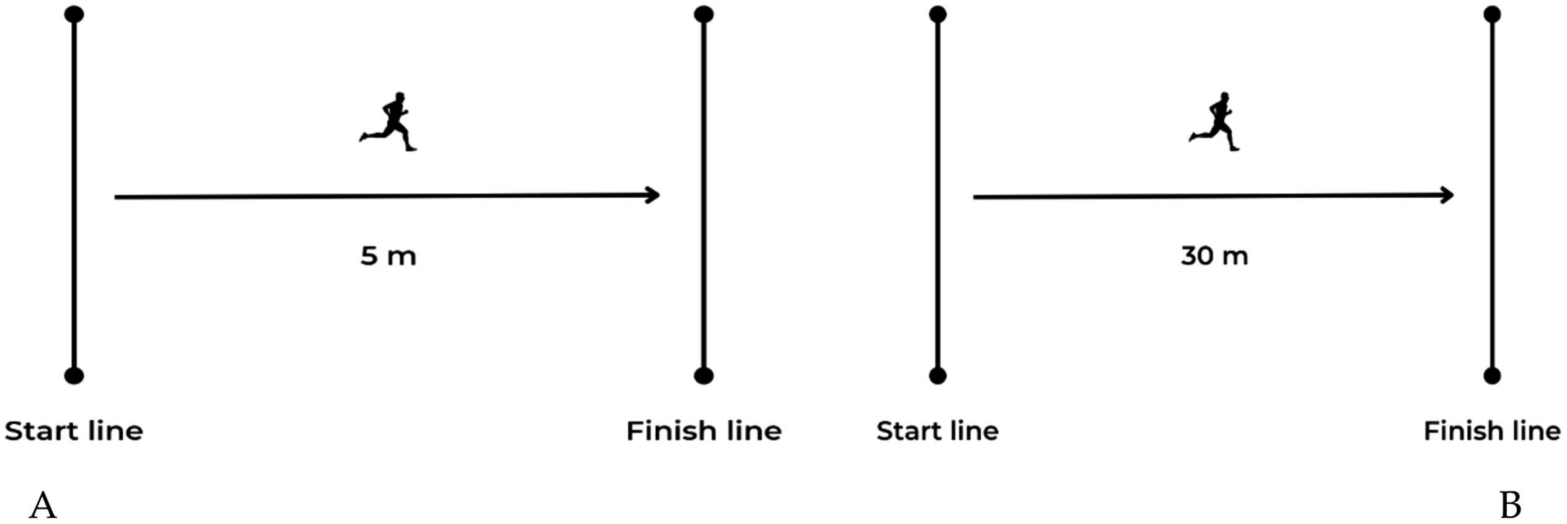
Schematic presentation of the 5 m (A) and 30 m (B) sprint test. Circles represent the position of photocells.

The running times were recorded by two pairs of dual-beam Witty Gate photocells (Microgate, Bolzano, Italy). Following the warm-up phase, participants executed two successive 30-m sprints with a 5-min rest interval in between the trials (Figures 2A,B). To avert premature activation of the starting gate, participants commenced with their leading foot positioned 0.5 m behind the initial timing gate. The best time from the two trials, both at 5 and 30 m, was preserved for further analysis.

### 2.4 Change of direction test

**Figure 3:**
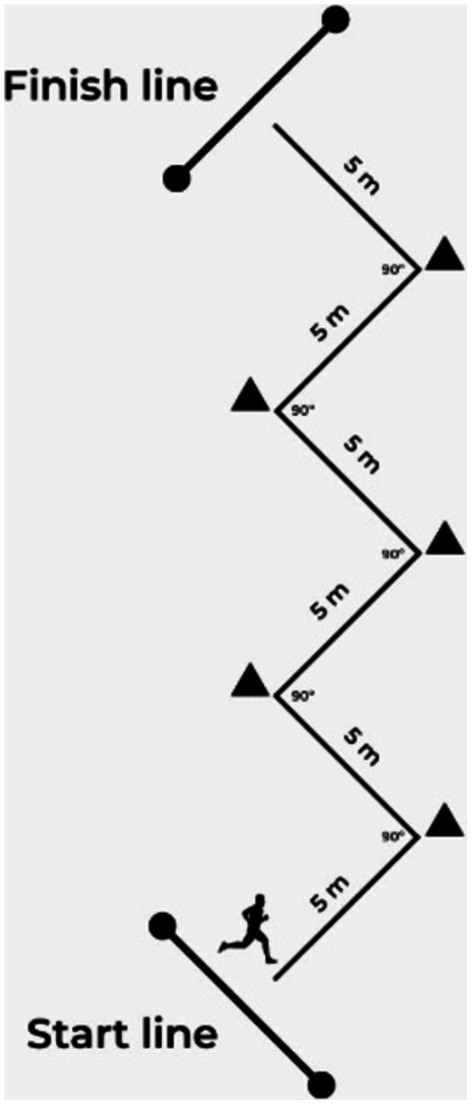
Schematic presentation of the 90° change of direction test. Circles represent the position of photocells.

After the linear sprint test, participants rested for 5 min and then performed the COD tests (Figure 3). Each participant performed tests with a 90° COD (ZigZag test). The participant’s task’ was to cover a 30 m section with designated cones with changes in direction and proper movement pattern (44). The running test was performed twice with a 5-min rest interval between attempts. The fastest time from each COD test was retained for further analysis.

### 2.5 Change of direction deficits

The COD deficit was calculated by the difference in run time in 30 m COD test [s] and 30 m linear sprint time.

### 2.6 Vitamin D serum analysis

Serum 25OH-Vitamin D was determined by RIA-CT KIP1971/KIP1974 (DIAsource ImmunoAssays SA, Louvain-la-Neuve, Belgium). For statistical analysis participants were divided into two groups according to the concentration of 25(OH)D: inadequate levels of 25(OH)D (vitamin D deficiency/insufficiency, <30 mg/mL), and adequate levels of 25(OH)D (vitamin D sufficiency, >30 mg/mL).

### 2.7 Statistical analysis

All statistical analyses were performed using Statistica 13.1. The Shapiro–Wilk test was used in order to verify the normality of the distribution. Pearson’s correlation and regression analysis were used to analyze the relationships between variables. Student’s t-test was used to verify the significance of differences for paired values. For pairwise comparisons, effect sizes were determined by Cohen’s d which was characterized as large (d > 0.8), moderate (d between 0.8 and 0.5), small (d between 0.49 and 0.20) and trivial (d < 0.05). Percent changes with 95% confidence intervals (95CI) were also calculated. Statistical significance was set at *p* < 0.05.

## 3 Results

**Figure 4:**
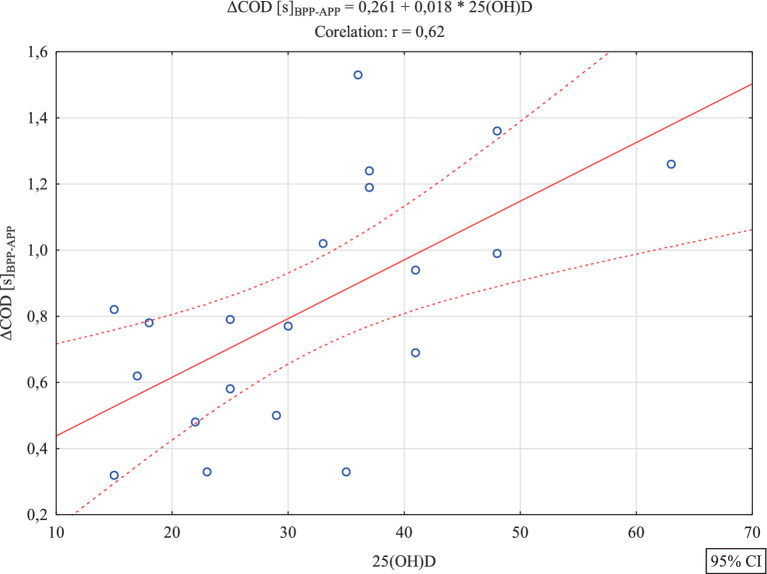
Scatterplot with regression function for the analyzed variables 25(OH)D and ΔCOD [s]_BPP-APP._

**Figure 5:**
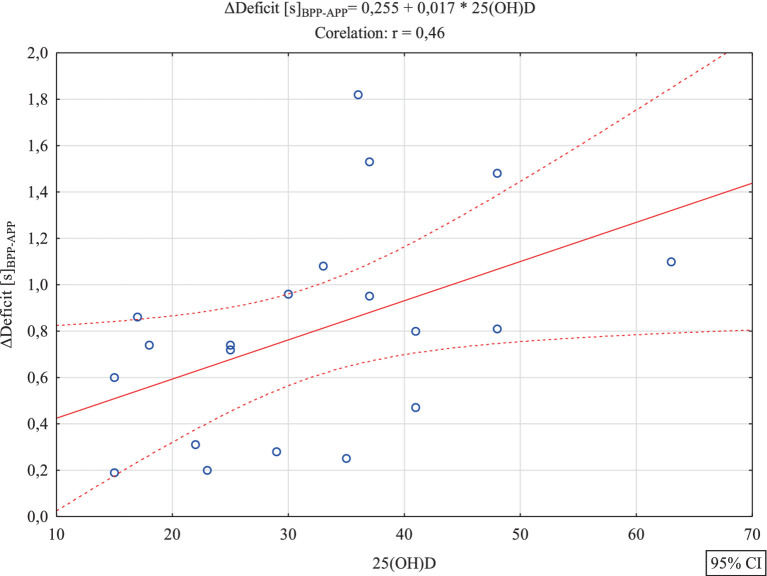
Scatterplot with regression function for the analyzed variables 25(OH)D and ΔDeficit [s]_BPP-APP._

Table 1 presents basic descriptive statistics for the analyzed variables. Further analysis aimed to verify whether there were significant correlations between the level of vitamin D and variables illustrating the effects of the experiment. Between 25(OH)D and Δ5 m [s]_BPP-APP_ R = 0.12; *p* = 0.61 and between 25(OH)D and Δ30 m [s]_BPP-APP_ R = 0.06; *p* = 0.81 no significant correlations were found. Statistically significant correlations were found between vitamin D level and the effect of the experiment in the case of the variables ΔCOD [s]_BPP-APP_ R = 0.62; *p* = 0.0035 (strong correlation) and ΔDeficit [s]_BPP-APP_ R = 0.46; *p* = 0.039 (average correlation).It was found that the increasing trend was also statistically significant *F*(1,18) = 13.25, *p* < 0.0018, SE = 0.28 and ΔDeficit [s]_BPP-APP_ R = 0.46, *p* = 0.039 (average correlation). It was also found that the increasing trend was statistically significant F(1,18) = 6.91, *p* < 0.017, SE = 0.39. In both cases, positive correlations were found, which indicates that with an increase in the concentration of vitamin D, a significantly higher training effect was found, i.e., a improvement of the test time in the case of COD and a reduction in the size of the deficit. These results are confirmed by Figures 4, 5 (see Table 2).

**Table 1:** Basic descriptive statistics for the analyzed variables.

Variable	M	SD	−95%CI	95%CI	Me	Min	Max
5 m [s] – BPP	1.05	0.07	1.02	1.08	1.06	0.92	1.18
5 m [s] – APP	1.02	0.06	0.99	1.04	1.01	0.94	1.12
30 m [s] – BPP	4.16	0.14	4.10	4.23	4.16	3.89	4.47
30 m [s] – APP	4.13	0.19	4.04	4.22	4.08	3.91	4.50
COD [s] – BPP	6.12	0.26	5.99	6.24	6.16	5.60	6.48
COD [s] – APP	5.29	0.34	5.13	5.45	5.13	4.92	5.99
DEFICIT [s] – BPP	1.96	0.29	1.82	2.09	1.93	1.40	2.50
DEFICIT [s] – APP	1.16	0.32	1.01	1.31	1.14	0.54	1.72
Δ5 m [s]_BPP-APP_	0.031	0.093	−0.012	0.074	0.045	−0.140	0.180
Δ30 m [s]_BPP-APP_	0.032	0.182	−0.053	0.118	0.105	−0.290	0.240
ΔCOD [s]_BPP-APP_	0.827	0.359	0.659	0.995	0.785	0.320	1.530
ΔDeficit [s]_BPP-APP_	0.795	0.457	0.581	1.008	0.770	0.190	1.820
25(OH)D	31.90	12.57	26.02	37.78	31.50	15.00	63.00
Δ5 m [s]_BPP-APP_ 25(OH)D ≤ 30	0.027	0.061	−0.017	0.071	0.045	−0.080	0.120
Δ5 m [s]_BPP-APP_ 25(OH)D > 30	0.035	0.119	−0.050	0.120	0.050	−0.140	0.180
Δ30 m [s]_BPP-APP_ 25(OH)D ≤ 30	0.039	0.171	−0.083	0.161	0.090	−0.240	0.220
Δ30 m [s]_BPP-APP_ 25(OH)D > 30	0.026	0.203	−0.119	0.171	0.110	−0.290	0.240
ΔCOD [s]_BPP-APP_ 25(OH)D ≤ 30	0.599	0.189	0.464	0.734	0.600	0.320	0.820
ΔCOD [s]_BPP-APP_ (OH)D > 30	1.055	0.348	0.806	1.304	1.105	0.330	1.530
ΔDeficit [s]_BPP-APP_ 25(OH)D ≤ 30	0.560	0.289	0.354	0.766	0.660	0.190	0.960
ΔDeficit [s]_BPP-APP_ 25(OH)D > 30	1.029	0.485	0.682	1.376	1.015	0.250	1.820

M, mean; SD, standard deviation; 95%CI, confidence interval; Me, median; BPP, before the preparation period of the summer season; APP, after the preparation period of the summer season.

**Table 2:** Pearson’s correlation 25(OH)D and dependent variables.

25(OH)D	r(X,Y)	*r* ^2^	*t*	*p*
Δ5 m _BPP-APP_	0.12	0.01	0.51	0.61
Δ30 m _BPP-APP_	0.06	0.00	0.25	0.81
ΔCOD _BPP-APP_	0.62	0.39	3.36	0.0035
ΔDeficit _BPP-APP_	0.46	0.22	2.23	0.039

**Figure 6:**
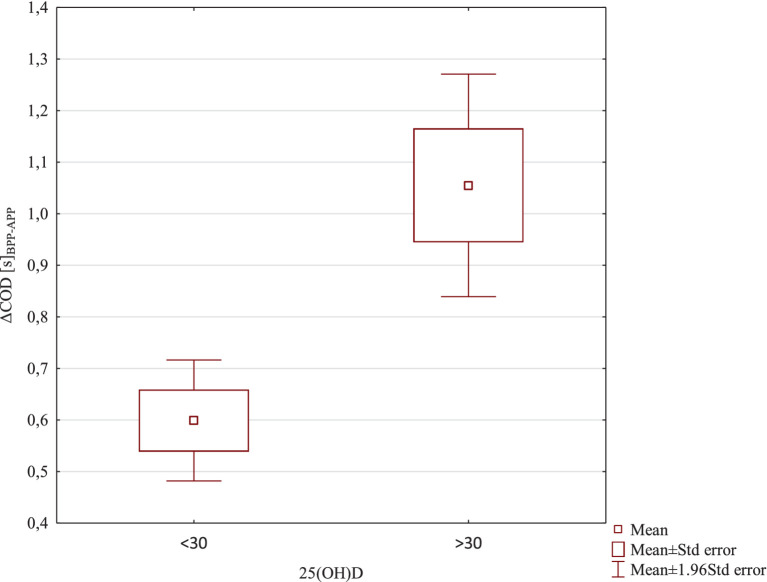
ΔCOD [s]_BPP-APP_ results due to 25(OH)D levels.

**Figure 7:**
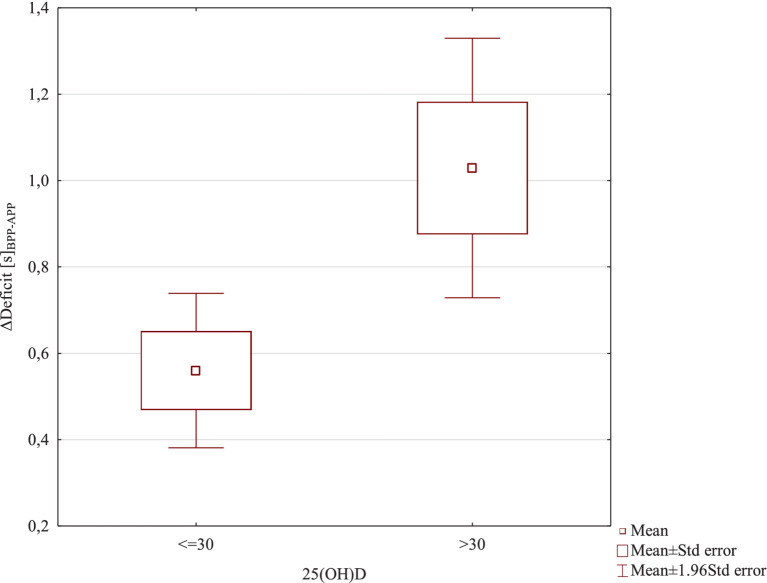
ΔDeficit [s]_BPP-APP_ results due to 25(OH)D levels.

In further analyses, it was verified whether the level of 25(OH)D ≤ 30 or 25(OH)D > 30 significantly differentiated the results of training effects. For this purpose, the Student’s *t* test for independent samples was used. The level of 25(OH)D ≤ 30 or 25(OH)D > 30 did not significantly differentiate training effects for the variables Δ5 m [s]_BPP-APP_ T = −0.19; df = 18; *p* = 0.85 and Δ30 m [s]_BPP-APP_ T = 0.15; df = 18; *p* = 0.87. A significantly higher training effect ΔCOD [s]_BPP-APP_ was found in the 25(OH)D > 30 group M = 1.055 ± 0.35; compared to the 25(OH)D group ≤30 M = 0.60 ± 0.19; T = −3.64; df = 18; *p* = 0.0019; d = 1.63. A significantly higher training effect was also found in case of the ΔDeficit [s]_BPP-APP_ in the 25(OH)D > 30 group M = 1.029 ± 0.49; compared to the 25(OH)D group ≤30 M = 0.56 ± 0.29; T = −2.63; df = 18; *p* = 0.017; d = 1.13. These results are also confirmed by Figures 6, 7.

## 4 Discussion

In our study, we focused on two main aspects. Firstly, we measured the levels of 25(OH)D in soccer players’ serum during winter and compared the number of participants with insufficient and sufficient concentrations. The results revealed that 50% of soccer players had an insufficient level or deficit of 25(OH)D (≤ 30 ng/mL), with 20% having a deficiency of 25(OH)D (<20 ng/mL) and 30% having insufficient 25(OH)D levels (between 20–30 ng/mL). The remaining 50% had sufficient 25(OH)D levels (> 30 ng/mL).

Similar results of serum vitamin D concentration, as measured in our study, were also reported by other authors (20, 22, 45, 47). Gilic et al. (20) found that among the studied youth Croatia soccer players, 54% of 52 had 25(OH)D insufficient concentrations, even though they were living at a southern latitude. Bezuglov et al. (32) reported low 25(OH)D levels in 42.8% of 131 youth Russian soccer players residing in Moscow at a north latitude of 55.9°. Bezuglov and colleagues (32) explained this by lower training loads during winter compared to the summer.

In our previous study (22), we observed that during winter, 12 out of 28 soccer players had 25(OH)D serum concentrations below 20 ng/mL, and 14 out of 28 had concentrations below 30 ng/mL. Jastrzebska and co-authors (47) observed lower 25(OH)D concentration during periods of low sunlight exposure with 24 young soccer players. Conversely, Kondurakis et al. (45) observed significantly higher vitamin D concentrations following the six-week off-season period.

These results align with other studies measuring vitamin D concentration in soccer players, indicating a high prevalence of insufficiency and deficiency even in regions with sufficient sunlight (22, 28, 32, 33, 45, 48). In addition to disturbed skin synthesis in the winter, authors suggest that intense soccer training affects vitamin D levels (31, 49). Morton et al. (31) observed much lower vitamin D levels during training periods compared to the six-week detraining period, despite the fact that both periods happened in the summer (31, 49). Kondurakis et al. (45) proposed that intense training sessions, by inducing stress, weaken the athletes’ immune system and play a regulatory role in vitamin D levels (45, 50, 51). They supported their hypothesis by testing soccer players during the pre-season and at the beginning of the season, periods of high training and frequent soccer matches (45). Vitamin D status is strongly associated with geographic location, season, outdoor or indoor training, and the intensity of training (27, 32, 52, 53). According to recent scientific reports, the increased utilization of 25(OH)D by cells of the immune system for the synthesis of defence substances against stress induced by daily high training loads is a crucial factor (19, 31, 50). Additionally, during winter, immune cells use vitamin D more frequently due to the particularly intense period of infections (19).

### 4.1 Vitamin D and performance

In the second aspect of our study, we investigated whether serum concentration of 25(OH)D affects speed and change of direction performance (COD) in professional soccer players. The main finding was that serum 25(OH)D concentration did not correlate with 5 m and 30 m linear sprint times, but it did correlate with COD performance. Comparisons between the level of 25(OH)D and Δ5 m [s] before-after and between 25(OH)D and Δ30 m [s] before-after did not reveal any significant associations. Significant correlations were found between the level of vitamin D and the effect of training on ΔCOD [s] before-after (*p* = 0.0035) (strong correlation) and Δdeficit [s] before-after (*p* = 0.039) (average correlation). In both cases, positive correlations were found, indicating that with an increase in the level of vitamin D, a significantly higher training effect was found. This resulted in a improvement of COD performance and a reduction in the deficit value. We also examined whether soccer players with a vitamin D level below 30 ng/mL (insufficient concentration) and those with a level above 30 ng/mL (sufficient concentration) experienced the same training effects and achieved similar results in the speed and COD tests. The level of 25(OH)D ≤ 30 or 25(OH)D > 30 did not significantly differentiate training effects for the variables Δ5 m [s] before-after and Δ30 m [s] before-after. However, a significantly higher training effect in the ΔCOD [s] before-after was found in soccer players with 25(OH)D > 30 compared to soccer players with 25(OH)D ≤ 30 (*p* = 0.0019). Also, a significantly higher training effect was found in case of Δdeficit [s] before-after in soccer players with 25(OH)D > 30 compared to soccer players with 25(OH)D ≤ 30 (*p* = 0.017). The results presented by other authors on the effect of vitamin D levels on performance are inconsistent. Most researchers studying soccer players confirm a positive correlation between vitamin D levels and speed and strength (20, 22, 27, 42, 45). In Gilic et al.’s (20) study with 52 young Croatian soccer players, better results were observed in speed tests over distances of 10 m and 20 m, as well as in COD tests in players with higher 25(OH)D. In our previous study (22) with 28 soccer players, we observed that the level of vitamin D at different times of the year influenced the results of speed tests over distances of 5 m and 30 m. Significant differences in 25(OH)D and 5 m speed test results were observed following summer compared with winter. Our results are consistent with the data presented by Jastrzebska et al. (42), Koundourakis et al. (45), and Książek et al. (30). In our research, similar to Jastrzebska et al. (42), soccer players with higher concentrations of 25(OH)D achieved better results in COD tests. They also achieved significantly better results on the deficit variable, which determines the differences in time over a distance of 30 m in a straight line and 30 m in a zig-zag manner. Unlike in our research, Jastrzebska and colleagues (42) found significant positive correlations between 25(OH)D concentration and sprint results over distances of 10 m and 30 m. The authors confirmed that players with a higher vitamin D level reached better results in the speed tests. They explained this by stating that the vitamin D resources stored in athletes’ bodies effectively influence the level of their anaerobic fitness. However, they concluded that it is still unclear whether the observed changes are due to differences in 25(OH)D concentration or applied training loads. In their opinion, the changes in speed and explosive power demonstrated in their study were caused by the applied training loads rather than by changes in 25(OH)D concentration. In turn, Skalska et al. (54) suggested that a higher 25(OH)D concentration with the same training load can effectively improve the level of anaerobic fitness. Also, Książek and colleagues (29) determined the relationship between 25-(OH)D concentration and performance in 24 soccer players. These authors showed a significant association between 3-epi-25-(OH)D3 and handgrip strength and vertical jump variables in soccer players and concluded that vitamin D metabolites might be involved in skeletal muscle function (18, 55). Contrary to the previous study, some authors did not confirm an association between vitamin D serum concentrations and strength performance in soccer players (5, 29). Książek et al. (29) did not find any correlations between 25(OH)D concentration and muscle strength or maximum oxygen uptake in Polish youth soccer players. Also, Branstrom et al. (5) in a study with Swedish female soccer players did not confirm a correlation between 25(OH)D and muscle performance assessed through isokinetic knee extension and flexion, countermovement jump, and sprint running.

Mechanisms by which vitamin D can influence speed performance are still hypothetical (20, 34, 36). Vitamin D controls and regulates the expression of muscle proteins and other cellular proteins synthesis, which are involved in calcium signaling and phosphate-dependent cellular metabolism, including energy resynthesis from ATP and phosphocreatine (34, 38, 56). Also, vitamin D influences muscle cellular calcium concentrations, which directly impact muscle contraction (19, 57). Vitamin D increases the influx of calcium into the cytoplasm by activating cellular kinases, empowering calcium to bind to the troponin-tropomyosin complex, resulting in exposure to active binding sites and allowing muscle contraction (20, 58). The more calcium ions are released into the cytoplasmic area, the more efficient the movement of myosin fibers through actin filaments, which may result in greater muscle contractile force and directly impact results in sprint and change of direction performance (9, 56). Additionally, VDR receptors present in myofibrils suggest a vitamin D genomic effect in their area by increasing the synthesis of muscle fibers (57). Vitamin D influences the size of fast-twitch fibers (37), which are important for the explosive type of human movement. Fast-twitch muscles can increase the muscle’s ability to generate energy very fast, resulting in higher speed and better neuromuscular coordination (20, 59). Vitamin D also increases the expression of IGF-1, which has an important role in muscle remodeling and hypertrophy (31). It is also worth adding that a higher level of vitamin D affects the synthesis of testosterone, which increases the synthesis of muscle proteins and indirectly affects muscle cellular metabolism and, finally, muscular speed and strength (9, 21, 37).

Due to the high use of vitamin D by athletes’ bodies every day, its level should be monitored throughout the year (17, 22). In the case of football players whose blood tests show a deficit or low level of 25(OH)D, it should be supplemented (32, 45). Our results confirmed previous studies that in soccers whose 25(OH)D level was above 30 ng/mL at the beginning of the preparatory period, training loads significantly improved speed (22, 32, 45). However, much research still needs to be done in this area to clearly confirm our results.

Our study has several limitations. Firstly, the absence of a control group impacts the robustness of our findings. Additionally, we did not measure dietary vitamin D intake and serum vitamin D concentration after the preparatory period. The study also did not assess other molecules such as 1.25(OH)D, Vitamin D Binding Protein (VDBP), or molecular variables such as vitamin D receptor (VDR) polymorphism or its mutations. These omissions limit the accuracy of interpreting potential mechanisms influencing vitamin D’s impact on performance. Furthermore, the study’s small sample size, limited to professional soccer players, restricts the generalizability of our results to other sports. Exploring the effects of various training stresses on vitamin D status could yield interesting insights but was not addressed in this study. In turn the strengths of our study include the research group, which consisted of professional Premier League soccers, and the project’s assumptions, which require minimal financial resources and are easy to implement by coaches in most soccer clubs. In the future, we plan to conduct similar studies with other groups of athletes, incorporating vitamin D supplementation.

## 5 Conclusion

In summary, we conclude that participants with higher 25(OH)D levels achieved superior results in the COD test and demonstrated better outcomes in the deficit measures, affirming the positive influence of 25(OH)D on muscle metabolism. The practical implications of our findings suggest that low vitamin D levels may impair muscle function, which could be reflected in poorer performance test results.

## Data Availability

The original contributions presented in the study are included in the article/supplementary material, further inquiries can be directed to the corresponding author.
